# Different and Similar at the Same Time. Cultural Competence through the Leans of Healthcare Providers

**DOI:** 10.3389/fpsyg.2017.01426

**Published:** 2017-08-22

**Authors:** Giuseppina Dell’Aversana, Andreina Bruno

**Affiliations:** Department of Educational Sciences, University of Genoa Genoa, Italy

**Keywords:** cultural competence, diversity management, health services, migrant, inequalities

## Abstract

Cultural competence (CC) for professionals and organizations has been recognized as a key strategy to reduce health care inequalities for migrants and to promote responsiveness to diversity. For decades its main aim has been matching health services to the cultural needs of migrant users. Otherwise literature highlighted the need to find a pragmatic middle way between the ‘static’ and the ‘dynamic’ views of culture that are recognizable in CC approaches. A pragmatic middle way to CC will be proposed as the way to respect diversity, even responding to cultural issues, without stereotyping or discriminating. To understand conditions that favor this pragmatic middle way this study aims to explore: (1) perceptions of healthcare providers in managing diversity; (2) strategies used to meet health needs at a professional and organizational level. A qualitative case study was conducted in a healthcare service renowned for its engagement in migrant sensitive care. Four different professional figures involved in CC strategies at different levels, both managerial and non-managerial, were interviewed. Data were analyzed using thematic analysis. Findings indicated that dealing with diversity poses challenges for healthcare providers, by confronting them with multilevel barriers to quality of care. A pragmatic middle way to CC seems to rely on complex understanding of the interaction between patients social conditions and the capacity of the institutional system to promote equity. Professional and organizational strategies, such as inter-professional and intersectional collaboration, cultural food adaptation and professional training can enhance quality of care, patient compliance responding to social and cultural needs.

## Introduction

According to International Organization for Migration, the estimated total number of international migrants has reached 244 million in 2015 (International Organization for Migration [IOM], 2016). In Europe, which has seen a consistent rise in the trend of migration since 2005, migrants are 76 million and constitute almost 9% of the total European population.

The evidence that inequities exist in the field of migrant health has demanded for a reorientation of health policies to better protect migrants’ health and provision of equitable health services. In 2008, The World Health Organization called for migrant sensitive health policies, practices and health systems with the WHR61.17 Resolution on the ‘Health of migrants’ (World Health Organization, 2008). The utilization of health services by migrants differs from that by non-migrants, as both migrants’ needs and their access to health care are affected by a number of factors related to the process of migration, including health and socioeconomic status, self-perceived needs, health beliefs, health-seeking behavior, language barriers, cultural differences, trauma and newness (Norredam and Krasnik, 2011). In addition, migrants face legal obstacles in most countries when accessing health care and may receive poorer service quality for discriminative attitudes of health staff, impacting diagnostics, medication, medical follow-up, hospital visits and admission, as well as patients’ adherence to treatment.

A key concept in this field is cultural competence (CC) for professionals and organizations, defined as: “a set of congruent behaviors, attitudes, and policies, that come together in a system, agency, or among professionals, and enable effective work in cross-cultural situations” (Cross et al., 1989, p. 28). Good practices in culturally competent health care include the training of staff, diversification of the workforce, use of cultural mediators, and adaptation of protocols, procedures and treatment methods (Office of Minority Health [OMH], 2001, 2013; Fernandes and Pereira Miguel, 2009; Mladovsky et al., 2012).

For decades the main aim of CC has been matching health services to the needs of migrant users, to bridge ‘cultural gaps’ (Ingleby, 2011). Social science criticized CC discourse by highlighting the lack of conceptual clarity around the use of the term ‘culture’ in clinical encounters and inadequate recognition of the ‘culture of medicine’ (Thackrah and Thompson, 2013). Narrow or static concepts of culture often conflate culture with race and ethnicity: they fail to capture diversity within groups and reduce the effectiveness of CC strategies by stereotyping health needs. Thus using culture as a concept to reduce health inequalities for ‘diverse’ groups entails risks which include categorization and overestimation of cultural dimensions at the expense of social, political and biographical ones (Kleinman and Benson, 2006).

Partly as a result of this criticism, the concept of culture assumed by researchers on health services has changed over time. Emphasis on its dynamic dimension permits to recognize that CC involves skills in intercultural communication, attitudes of respect and openness, and relevant knowledge (Ingleby, 2011). Another important dimension is professionals’ self-assessment of their own culture and implicit assumptions. In this way many aspects of the CC formulation are also central aspects of patient centeredness (Saha et al., 2008). However, the dynamic conceptualization of culture has the advantage of reducing the risk of stereotyping medical practices but, at the same time, the disadvantage of limiting the ability of health services to focus on the needs of particular groups.

Since the cultural dimension in clinical practice is central, as health and disease experiences are inter-subjectively defined in symbolic exchanges (Scheper-Hughes and Lock, 1987), according to Ingleby (2011, p. 236) “between the ‘static’ and the ‘dynamic’ views of culture a pragmatic middle way has to be found.” The ‘pragmatic middle way’ permits to respect diversity, even responding to cultural issues, without stereotyping or discriminating. In fact, fear of discrimination involved by categorization is often presented as a dilemma which limits implementation of culturally competent strategies (Seeleman et al., 2015; Suphanchaimat et al., 2015), that remain patchy widespread in Europe.

Following Ingleby’s suggestion, in our proposal we will examine, through a case study, how the pragmatic middle way is sustainable and may permit either to include the advantages and either to minimize the disadvantages of the ‘static’ and ‘dynamic’ approaches, above discussed. To understand conditions that favor the pragmatic middle way this study aims to: (1) investigate perceptions of healthcare providers in managing diversity, as well as examine the barriers faced in their practices; (2) identify strategies used to meet health needs at a professional and organizational level.

## Method

The study was conducted in an infectious diseases department of a hospital with a high percentage of migrant users, located in a suburban area in France renowned for its past and current engagement in migrant sensitive care. Semi-structured in-depth interviews were conducted in 2013 with four health providers who represented different professional figures at different levels, both managerial and non-managerial: a cultural mediator, a psychologist, the head physician and a psychiatric nurse. Participants were 3 women and 1 man: 3 are French and 1 has African origins. The service and 3 participants were chosen for their involvement in the Migrant Friendly Hospital Project, an European network dealing with migrant sensitive care (Migrant Friendly Hospitals, 2004) and for their expertise in cultural competent strategies. All subjects gave written informed consent and authorized and approved the use of anonymous data for publications.

An interview schedule was developed using open-ended questions. During face-to-face interviews participants were asked to describe priority health needs, the challenges of working with migrant patients and the strategies developed to overcome them. The average length of interviews was 60 min (minimum 45 – maximum 75). The interviews were transcribed verbatim and analyzed according to a data-led approach to maximize discovery and exploration (Braun and Clarke, 2006). The relevant material was selected and was subsequently encoded according to themes emerging from the data set. Specifically, our analysis involved different and iterative steps: data familiarization, initial coding generation, search for themes based on initial coding, review of themes, theme definition and labeling. Identified themes captured important aspects of the data in relation to the research questions. The credibility of analysis, as criterion for the qualitative research, was assessed through supervision sessions to check the coding strategies and to review the interpretation of the data, by discussing any reason of variation (Barbour, 2001). Rival configurations of themes were ultimately modified (Patton, 2002).

## Results

The first section focuses on challenges that emerge from dealing with and understanding diversity, while the second one focuses on organizational and professional responses.

### Challenges in Healthcare Provision

#### Dealing with Diversity

Diversity is a daily and structural dimension of the clinical service environment. When we asked to describe what challenges emerge in the provision of healthcare services for migrants, interviewees stated that practices were mainly influenced by different barriers: not only diverse cultural beliefs and language differences, but also legal, social, psychological, and relational aspects connected to the migrant status.

For many patients hospitalization is the first chance to obtain a legal document for healthcare access. Relational asymmetry is exacerbated by the distance of migrant patients from biomedical thought and practices that require patients to maintain a state of passivity and rely on the superiority of expert knowledge. Thus, cultural and political dimensions enter into the clinical field and in the relationship between provider and patient giving rise to patient anxieties. Refusal of treatment (i.e., food, magnetic resonance, blood sampling) recurs in interviews as a significant example of patient cultural needs and low health literacy that are often at the origin of conflicts and misunderstandings. In particular, discrimination and marginalization are highlighted in relation to the weight of the stigma affecting those suffering from infectious diseases, which cause isolation, delay in care and mistrust in the relationship with the institution and with providers.

*I remember a patient, we suspected tuberculosis and so we put him in isolation, after it turned out that he didn’t have tuberculosis. He was so angry, because for him it was discrimination, because he was black … You see, these little things, after explaining that it was a path that had to be explored, that fortunately he did not have TB, but for him we were racist because he was poor and black*. (1)

Dealing with diversity also means conflicts with organizational cultures tied to the bureaucratic model. An institutional culture that stresses cost efficiency, time pressure and standardized processes can limit the meeting of migrant health needs. These values can make providers interpret access regulation narrowly or fail to use interpreting services to reduce professional and organizational overload.

#### Understanding Diversity

The stories of respondents on their professional practice with diversity suggest a way of thinking about the migrant patient as having special needs and, at the same time, universal needs. In this way, diversity can be managed and assumed as the dialectic between universal and particular (Fassin, 2000).

*Migrants have exacerbated suffering because of migration, but there are other patients with serious suffering too*. (1)

*Migrant patients refuse treatment, but there are good ‘Franco- French’ people that do the same*. (4)

One interviewee in particular discusses the risks of a static vision of culture and of the definition of diversity in cultural terms:

*Culture; and I said human being: a ‘Franco-French,’ he is in a culture, isn’t he? Also in a family history, and even there he is different from his sisters and brothers, and at the same time similar. For foreign people, it’s not different! For me, bonding someone to his/her culture is a racist discussion*. (1)

From an organizational point of view, the principle of justice in care leads to respect for diversity and responsiveness of medical processes and is interpreted as the attainment of the same level for those that have less. In addition, the quality of taking charge of migrant patients is considered an excellent indicator for that of each patient. These values are the basis to develop professional and organizational strategies, described in the next sections.

### Health Service Responses

#### Professional Strategies

To provide health services that respond to the above challenges, the relational strategies of practitioners deal with the ‘sense of otherness.’ Basic knowledge of other cultural representations is recognized as an important attribute of providers, but there are primarily attitudes of openness, acceptance of doubt, a more participative communicative style, and relational skills. Respect for patients’ values, preferences and expressed needs is important for every patient.

*Since providers are fixed in their own cultural model and they can’t imagine or accept those who have a different model and that people can react differently to what they expect, it can’t work. As you have this ‘sense of otherness’, the acknowledgment of diversity… everything is possible. It means adjusting practices, showing curiosity for ways of life… in sum the provider is primarily interested in the person instead of the disease, a unique person with their own peculiarities….this may change the relationship completely… the person may feel like they are accepted with their peculiarities, even the cultural ones. And trust and compliance become easy…. To treat a migrant you don’t have to be an anthropologist*. (3)

#### Organizational Strategies

To develop culturally competent services, recurrent strategies are utilized, as follows.

##### Involvement of different stakeholders and integrative functions

To enhance the quality of interventions, organizational inconsistencies are used as a source of information, notably through conflict understanding. For example, the preparation of ‘traditional’ food agreed with each patient, even of non-immigrant origin, is used as a lever for care quality during hospitalization. The cultural adaptation of food arises from an organizational criticality. Patients, who have already had severe weight loss caused by HIV, were worsening their condition by refusing served food and asking to be discharged. This could endanger treatment continuity given their often precarious social situation. This project required creating a network to connect patients and different providers: the psychologist who created the project, mediators, nurses, doctors, social workers. In particular, the mediator and the psychologist provide an integrative function, by promoting communication in cases of conflicts or misunderstandings, thanks to their interface position between patients and the medical and social staff.

*In the morning we contact the nurses or the head physician and the psychologist to know if there is someone else who needs the meal… Care assistants see how the patient eats. They speak to the staff and then the staff to us*. (2)

The network also involves external stakeholders: it is funded partly by an NGO and is based on collaboration with a mediator association. In addition, in order to hinder the various social factors of vulnerability, the staff collaborates with a day-care center near the hospital for HIV patients, which provides daily meals as well as literacy and health education.

##### Use of symbolic dimensions

The symbolic dimensions of disease are used during care to provide emotional support to patients. The mediators, who have different nationalities, eat with patients: the food is associated with a symbolism that recalls belonging and roots. At the same time sharing meals seems to reduce patient concern about stigma and isolation and to give relief by creating a moment for informal conversation in their native language.

*This reassured him about his HIV-positive status: it was possible to eat with him, without fear of being contaminated… there are all these dimensions here… and so we are ‘friendly.’* (1)

In the same way, additional strategies allow for a greater number of family members during visits and the involvement of the worshippers of the different religions represented in the hospital. Organizational practices are aimed not only at patient care, but also at staff training, as described in the last section.

##### Staff training regarding sensitivity to diversity

To support health staff in being sensitive to diversity (including the use of interpreting services), training sessions use clinical cases in which the cultural dimension is relevant. Moreover, the department organized staff exchanges with a Burundian hospital and voluntary weekly vacations in Africa to visit local hospitals and to attend traditional healing rituals. Lastly, an annual conference on ethno-cultural issues (e.g., about the symbolism of blood and death far from the land of origin) involves experts and associations, as an outcome of the MFH project.

These activities are stimulated by the head physician, who has an ethno-medical education and a key role in disseminating shared values and CC strategies. Although it is a priority, training has some areas of concern: difficulty in assessing the sensitivity of staff practices, high staff turnover and presence of countercultures.

*There are certain people who are particularly sensitive to this, others are almost neutral and finally there is a group that may be even a little oppositive: “why do we care so much about patients, especially migrants? The patient is there primarily to receive treatment.”* (3)

## Discussion and Conclusion

Dealing with diversity poses some challenges for healthcare providers in the clinical relationship and multiple barriers to quality care services. Barriers are understood in a multilevel perspective (Suphanchaimat et al., 2015): (1) At the level of interaction between healthcare providers and migrant patients, trust and the possibility of recognizing diversity are affected by the asymmetrical relationship embodied in political and historical racial relations; (2) At the level of interaction between healthcare providers and their workplace context, there are conflicts between bureaucratic and patient-centeredness organizational cultures; (3) At the level of influence of external factors, differences are between approaches to health and wellbeing.

There is a complex understanding of diversity as a result of the interaction between values, beliefs, political conditions and the capacity of the institutional system to promote equity. For respondents, the migrant patient is not perceived as a ‘distant’ category, closed in that absolute of the difference mentioned by Fassin (2000), that undermines habitual ways of thinking and acting. In particular, the pragmatic middle way seems to rely on the following values: (1) The principle of justice seems to balance the opposition between the principle of universalism (‘treating the equal equally’) and that of particularism (‘treating diversity differently’); (2) The uniqueness of the patient based on the recognition that ‘all people are equal and different at the same time’ emerges as a dialectic between universality and particularism (Fassin, 2000) leading to patient-centeredness; (3) Responses to cultural and social problems need to be related to disease and personal patient conditions.

An understanding of diversity built on a ‘sense of otherness’ overlaps patient-centeredness. It is comparable to that described by Fortin and Maynard (2015), who, between reifying and universalist views of diversity, found an approach called humanist, in which differences are contextualized. Whereas for the first, diversity is anchored to ethnic origins and tends to be interpreted as ‘deviance to be eliminated,’ the second one tends to ignore it in the name of the principle of universalism. Instead, physicians with a ‘humanist’ approach have a complex understanding of diversity and conflict is seen to lead to compromise and reconciliation between opposing actors. In this way CC for professionals deals with reflective practices to manage uncertainties and multiform aspects of the work (Bruno et al., 2011; Bruno and Dell’Aversana, 2017a,b,c).

The organizational culture enables the service to react constructively to organizational inconsistencies, using them as a way to innovate and learn (Bruno and Bracco, 2016). Organizational strategies, such as inter-professional and intersectional collaboration, cultural food adaptation and professional training are set up as a way to enhance quality of care, patient satisfaction and compliance responding to social and cultural needs. These different strategies help the service to recognize the symbolic aspects of the disease, create relationships of trust between providers and patients and a way to negotiate and prevent conflicts. Such devices can reduce discontinuity in care and in the practitioner–patient relationship and rectify the absence of well-defined channels of communication which are considered to be antecedent factors of conflict (Fortin and Maynard, 2015).

Another key point for services is to conceive them as a network, since the quality of care and of the relationship with patients is proportional to the care of the relationships between all involved actors. This also seems necessary to protect the sustainability of interventions and to obtain economic resources during economic crisis (Paris, 2014; Mucci et al., 2016). These findings put emphasis on the importance of promoting a shared culture of CC and assessment strategies to monitor and make the quality of care visible: leadership and training experiences can be used as a catalyst for cultural change and innovation, in order to enhance the quality of service for every patient.

## Ethics Statement

The interviews included a statement about personal data treatment, in accordance with the Italian privacy law (Law Decree DL-196/2003). Practitioners authorized the use of anonymous/collective data for scientific publications. The ethical approval was not sought, since practitioners approved the use of anonymous/collective data and the research is based on the study of psychosocial variables that refer to the work environment. For this reason, the study cannot be considered a medical research or an experiment on human subjects that need ethical approval following the Recommendations from WMA Declaration of Helsinki – Ethical Principles for Medical Research – Involving Human Subjects (World Medical Association [WMA], 2013).

## Author Contributions

GD and AB conceptualized the study and chose the theoretical framework. GD collected and analyzed the data. AB supervised interpretation of data revising it critically. AB and GD wrote, improved and revised the manuscript several times.

## Conflict of Interest Statement

The authors declare that the research was conducted in the absence of any commercial or financial relationships that could be construed as a potential conflict of interest.
